# Lone Pair and Unique N‐Bridging of Novel Titanium Nitridophosphate

**DOI:** 10.1002/advs.202412830

**Published:** 2025-02-11

**Authors:** Peter Ufondu, Sakshi, Teak D. Boyko, Monika M. Pointner, Wolfgang Schnick, Alexander Moewes

**Affiliations:** ^1^ Department of Physics and Engineering Physics University of Saskatchewan 116 Science Place Saskatoon Saskatchewan S7N 5E2 Canada; ^2^ Canadian Light Source 44 Innovation Blvd Saskatoon Saskatchewan S7N 2V3 Canada; ^3^ Department of Chemistry University of Munich (LMU) Butenandtstraße 5‐13 81377 Munich Germany

**Keywords:** DFT, LFMT, Lone pair, Nitride, Photovoltaic, RIXS

## Abstract

In exploring advanced materials for solar power, the novel titanium nitridophosphate (TiP_4_N_8_) stands out due to its unique linear nitrogen bridging. To investigate the elemental interactions responsible for the photovoltaic performance under visible light, the titanium *L*_2, 3_‐edges and nitrogen K‐edge are specifically explored using X‐ray absorption spectroscopy (XAS), X‐ray emission spectroscopy (XES), and resonant inelastic X‐ray scattering (RIXS) techniques to map the unoccupied and occupied electronic states. It is shown that the indirect interaction between the linear nitrogen bearing the lone pair and titanium is responsible for the bandgap of 1.55 ± 0.30 eV and 1.77 ± 0.30 eV in the β‐ and α‐TiP_4_N_8_ phases as well as the stability of the α‐phase. The formal oxidation state of the Ti ion in the β‐ and α‐phases are also validated to be trivalent (Ti^+3^) and both trivalent (Ti^+3^) and tetravalent (Ti^+4^), respectively.

## Graphical abstract

The analysis provides a comprehensive understanding of the structural stability and electronic factors that govern the bandgap, charge carrier mobility, and photovoltaic performance of novel titanium nitridophosphate (TiP_4_N_8_).

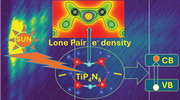

## 1 Introduction

The enigmatic nature of ternary transition metals‐bearing nitride compounds (TMNc) has been a captivating and extensively studied topic in materials science for the past few decades.^[^ ^1^ ^]^ Transition metal nitrides have long been recognized for their remarkable properties, including high hardness, exceptional chemical and thermal stability, and electronic versatility, which make them suitable for a range of applications from cutting tools to energy regeneration.^[^ ^2^, ^3^, ^4^, ^5^ ^]^ This consistent research interest is driven by the potential of TMNc to yield stable nitrides with suitable bandgaps for semiconductor applications, particularly in the fields of photovoltaics.^[^ ^6^, ^7^, ^8^, ^9^, ^10^, ^11^ ^]^ Despite this interest, the synthesis of TMNc containing phosphorus remains a significant challenge. Nonetheless, recent experimental reports have documented the successful synthesis of the first stable ternary titanium nitridophosphate (TiP_4_N_8_) through high‐pressure and high‐temperature methods.^[^ ^12^ ^]^ Characteristically, TiP_4_N_8_ exhibits a unique linear nitrogen bridging in the unit crystal structure that distinguishes it from other nitridophosphates.^[^ ^13^, ^14^ ^]^ The material's crystal structure was determined using X‐ray diffraction (XRD), which provided detailed insight into the atomic arrangement and bonding characteristics.^[^ ^12^ ^]^

Complementary to XRD, (scanning) transmission electron microscopy (TEM, STEM) and energy‐dispersive X‐ray spectroscopy (EDX) were employed to analyze the morphology and elemental composition of the two polymorphs α‐ and β‐TiP_4_N_8_ phase, respectively.^[^ ^12^ ^]^

Current findings highlight that TiP_4_N_8_ possesses excellent thermal stability,^[^ ^12^ ^]^ which is critical for its potential use in harsh environmental conditions typical for photovoltaic processes.

Additionally, preliminary studies using UV–vis spectroscopy and Vienna Ab initio Simulation Package (VASP) based on density functional theory (DFT) revealed that the material exhibits notable photovoltaic activity under visible light, which paves the way for its application in solar energy conversion and environmental remediation technologies.^[^ ^15^, ^16^, ^17^, ^18^ ^]^ Although these findings are significant, a comprehensive description of the mechanism mediating the reduced bandgap^[^ ^19^, ^20^, ^21^ ^]^ in TiP_4_N_8_ remains elusive. Understanding this mechanism is crucial for developing and optimizing TiP_4_N_8_ for effective use in photovoltaic devices. To investigate the elemental interaction responsible for the photovoltaic activity under visible light, we specifically explore the titanium *L*_2, 3_‐edges and nitrogen K‐edge using X‐ray absorption spectroscopy (XAS) to map the unoccupied electronic states, X‐ray emission spectroscopy (XES) and resonant inelastic X‐ray scattering (RIXS) techniques to probe the occupied electronic states, capturing detailed *dd* transitions, charge transfer excitations, and oxidation states.^[^ ^22^, ^23^ ^]^ The data obtained are further supported by integrating state‐of‐the‐art ligand field multiplet (LFM) and DFT calculations, refining our interpretation of the spectroscopic findings.

In this study, we validate the formal oxidation state of the titanium (Ti) ion within the two distinct polymorphs, α‐TiP_4_N_8_ and β‐TiP_4_N_8_. Through comprehensive analysis, we also elucidate the intricate role and indirect interactions of the lone electron pair associated with the linear bridging. These interactions are shown to significantly influence the semiconductor bandgap properties, particularly by modulating the electronic transitions within the ultraviolet (UV) region.

## 2 Results and Discussion

**Figure 1:**
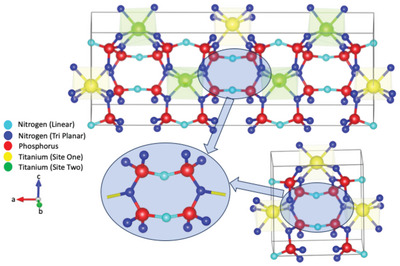
Crystallographic structure of β‐ (top), α‐TiP_4_N_8_ (bottom), showing the linear (light blue) and tri‐planar (dark blue) nitrogen bonding, titanium site one (yellow) and two (green) and the connector phosphorus (red) atoms. The magnified inset shows the presence of the linear nitrogen bridging in both phases.

**Figure** **1** shows the atomic arrangement of the two TiP_4_N_8_ phases studied here: β‐TiP_4_N_8_ (α‐TiP_4_N_8_) an amber red (ruby red) crystal annealed at 600 °C (700 °C). The β‐phase has two inequivalent titanium sites, site 1 (yellow) and site 2 (green), where, upon annealing the α‐phase is reduced to a smaller unit cell with one equivalent titanium site (yellow). The extended region shows the cyan‐colored linear nitrogen atoms bonded to two phosphorus atoms on opposite sides. It also features the blue tri‐planar nitrogen atoms bonded to a titanium atom. The synthesis details are described elsewhere.^[^ ^12^ ^]^

### 2.1 Ti L_2, 3_ −Edges Analysis

#### 2.1.1 β −Phase

**Figure 2:**
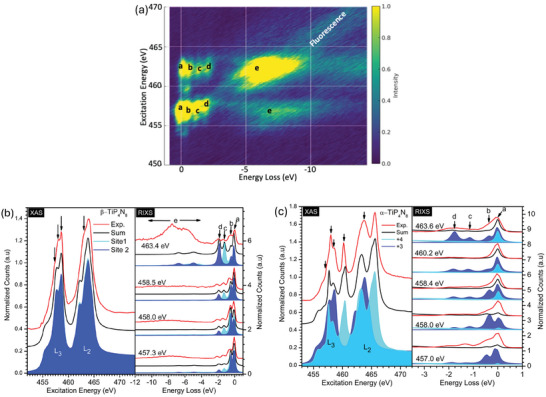
Panel (a) shows the 2D RIXS map of the β‐phase, plotting emitted photon count variations against energy loss on the *x*‐axis and the incident photon energy on the y‐axis. The color gradient in the map represents the intensity, with brighter regions indicating higher intensities. Highlighting *dd*, fluorescence, and charge transfer excitations. Panels (b) and (c) display the comparison of experimental (red line) and calculated (black line) X‐ray absorption spectroscopy (XAS) and resonant inelastic X‐ray scattering (RIXS) spectra for β‐ and α‐phases. In panel (b), the shaded blue and cyan spectra correspond to calculations with different equatorial distortion energy values. While in 2c, it corresponds to the Ti^+3^ and Ti^+4^ calculated spectra, respectively. The black arrows indicate the photon energies used for the RIXS measurements. The XAS and RIXS spectra have been normalized to the highest count feature.

**Figure** **2**a shows the Ti *L*_2, 3_‐edges RIXS map and exhibits three different contributions assigned to elastic scattering (*a*), inelastic scattering (*b*, *c*, *d*, and *e*), and fluorescence emission. Feature *a* is due to the elastic scattering off the sample (3*d* ^*n*^). Where *n* is the actual 3*d* occupation number of electrons. Features *b*, *c*, and *d* are the energy losses due to the reconfigured 3*d* electrons (3*d* ^n^*). Feature *b* is assigned by our calculations to the overlap ($d_{x^{2}y^{2}}$, d_*xy*_) (e′) orbital energy of Ti site 1 and site 2. Features *c* and *d* are assigned to (d_*xz*, *yz*_) (e″) orbital energies from site 1 and site 2, respectively. The photon counts of *c* and *d* at the *L*_3_‐edge are higher than at the *L*_2_‐edge, indicating a prominent decay mechanism. Feature *e* is the charge transfer Δ energy (energy difference between the centers of the 3*d* ^*n*^ and the 3*d* ^*n* + 1^ $\underline{L}$, where $\underline{L}$ denotes a ligand hole) and suggesting a constant energy loss features tracks with excitation energy. The hole at the 2*p*_1/2_ is populated by radiationless Coster‐Kronig^[^ ^24^ ^]^ transitions, which then subsequently decays (the hole) via fluorescence. Figure 2b displays the measured 2*p* XAS and 2*p*3*d* RIXS spectrum of the β‐phase at the Ti *L*_2, 3_‐edges, alongside the calculated spectra.

Figure 2b (left) shows the 2*p* ^5^3*d* ^1^ final state containing one 2*p* hole that strongly interacts with the 3*d* orbital (red spectrum). This gives rise to the final spectral shape. Due to the relatively low lifetime broadening for 2*p*, its spectral shapes can be observed experimentally. With its detailed multiplets, the spectrum splits into two energy levels, accounting for the total angular momentum *j*. This results in the *L*_2_ and *L*_3_ transition in the Ti 2*p* to 3*d* conduction band (CB) XAS spectrum. The energies of the final states are also affected by the 2*p*3*d* Coulomb and exchange interaction, crystal field splitting of the 3*d* orbital, and the 2*p* − 3*d* spin‐orbit coupling. The blue and cyan spectra are calculated with the same values for the crystal field distortion along the axial axis (Dν = −0.07 eV) and different values along the equatorial axis (Dμ) site 1 (0.5 eV) and site 2 (0.3 eV), to account for the change in bond lengths and bond angles. The resulting black spectrum is the combined ratio based on the numbers of titanium sites 1 (3) and 2 (4) polyhedra in a unit cell. The black calculated spectrum is compared with the red experimental measured spectrum. The black spectra reproduce the peak intensities and energy positions of the *L*_2_‐ and *L*_3_‐edges very well, showing that the converge parameters are correct. The small pre‐peaks marked by the first black arrow are related to multiplet splitting of the electron‐hole interactions and the crystal field symmetry. The spin‐orbit splitting parameters are scaled to their 3.40 eV and 0.0171 eV for the 2*p* and 3*d* orbitals, respectively, and the Slater–Condon parameters are 80% to 95% scaled to their Hatree–Fock values, resulting in almost the same intensities of the two edges. One aspect to note while comparing the two spectra is that the distortion along the axial direction does not affect the excited final state. However, the combined black spectrum agrees with our measured red spectrum. Upon comparison of the measured XAS spectrum with the published TiN spectrum^[^ ^19^, ^20^, ^21^, ^25^, ^26^ ^]^(not shown), we assign a formal oxidation state of +3 to Ti in the β‐phase, which contradicts the findings previously reported on the published synthesis,^[^ ^12^ ^]^ where Ti was found to be +4.

To further investigate the Ti valence, Figure 2b (right) shows RIXS spectra for four different photon excitation energies. At these photon excitation energies (457.3 eV, 458.0 eV, 458.5 eV, and 463.4 eV), the 2*p* electron is excited into various multiples of the 3*d* orbital. The strong Coulomb interaction binds it to the core hole, forming a core exciton. The core hole, therefore, is well‐screened, and the positive ion cores are unaffected. When the core hole is filled in the subsequent de‐excitation process, the 3*d* orbitals are rearranged to higher energy due to the energy loss to excitation within the 3*d* orbitals. This energy loss by each orbital is recorded in the form of a constant satellite in the low‐energy regions (*b*, *c*, *d*, and *e*). The presence of *dd* excitations *b*, *c*, and *d* at −0.52 eV, −1.33 eV, and −2.10 eV confirms the presence of an electron in the 3*d* orbital. The blue and cyan colors represent the 2*p*3*d* RIXS calculations for sites 1 and 2. Overall, the black spectrum for each excitation energy agrees very well with our measured spectra. Our LFM calculations confirm that feature *a* is a cumulative intensity of the e′ orbital from both Ti sites 1 and 2. Since the distortion in site 1 is greater compared to site 2, the 3*d* orbital splitting of the e″ is seen to have lower energy in the blue spectra (−2.10 eV) compared to the cyan spectra (−1.33 eV). The intensity of the broad charge transfer (Δ) feature *e* is poorly reproduced in the calculation due to the limitation of the LFM.^[^ ^27^ ^]^ From the calculation, the value of Δ converges at 3.9 eV for Ti sites 1 and 2, respectively. Also, the total number of electrons in the 3*d* orbital is approximately 1.25, which implies a charge transfer from the nitrogen ligand to the titanium to compensate for TiP_4_N_8_ ground state neutrality.

#### 2.1.2 α −Phase

Figure 2c shows the 2*p* XAS (453 − 472 eV) and 2*p*3*d* RIXS spectra obtained at the Ti *L*_2, 3_‐edges for α‐phase. The shape of the measured spectrum (red) is distinct from the Ti^+3^ β‐phase. There is a noticeable similarity between the red spectrum and titanium (IV) oxide (TiO_2_) spectrum^[^ ^28^ ^]^ (not shown here). The red spectrum features four distinct peaks, two each at the *L*_2_‐edge and the *L*_3_‐edge. Note that it has been confirmed that the formal oxidation of titanium in TiO_2_ is assigned Ti^+4^.^[^ ^28^ ^]^ By employing a linear combination of these Ti^+3^ and Ti^+4^ black spectra, we can compare and quantify the experimental data (depicted in red). Regarding energy and intensity, the experimental spectrum peaks are precisely replicated using the linearly combined Ti^+3^ and Ti^+4^ spectra. The comparison of the experimental and computed XAS spectrum indicates that the sample comprises 9.1% Ti^+3^ and 90.9% Ti^+4^. It is important to note that the cyan spectrum dominates the XAS spectrum with a charge ratio of 3.6, which agrees with the Bond‐Valence Sum (BVS) calculations.^[^ ^12^ ^]^ However, there is no report of the charge contribution from the 9.1% Ti^+3^ state.^[^ ^12^ ^]^ Our analysis leads to the conclusion that the sample comprises both trivalent and tetravalent titanium cations, with the chemical formula (${\mathrm{Ti}}_{0.09}^{+3}{\mathrm{Ti}}_{0.91}^{+4}$)P_4_N_8_. To ascertain the presence of Ti^+3^ state in the sample, we analyze the energy loss within the 3*d* orbitals. The parameters listed in Table S3 for Ti^+3^ and Ti^+4^ were applied to compute the XAS and their respective RIXS spectra simultaneously. These parameters accurately replicate the RIXS spectra for each excitation energy in α‐TiP_4_N_8_. Figure 2c (right) shows the measured RIXS and the corresponding LFM calculations. The 2*p* − 3*d* − 2*p* RIXS spectra at specific photon energies for the *L*_3_‐edge (456.35 eV, 458.40 eV, 460.35 eV) and *L*_2_‐edge (463.59 eV, 465.40 eV) ranges are presented. The higher energy loss features (fluorescence and charge transfer excitation regions) are identical to those of the β‐phase spectra and have been omitted. However, the charge transfer onset is shifted to a higher energy of 5.6 eV (see Figure S3) due to the strong indirect interaction by the titanium center and the N5 linear nitrogen ligand (Figure 4b). The general features labeled *a* to *d* on the RIXS spectra appear on the same energy scale as for the β‐phase RIXS spectra. However, their corresponding intensity is lower because of the low percentage of Ti^+3^ in the α‐TiP_4_N_8_. Again, there is excellent agreement between the calculation and measured RIXS spectra. This agreement arises from considering the presence of both Ti^+3^ and Ti^+4^. The low energy loss region is due to the presence of the Ti^+3^ state, with a *d* ^1^ configuration for Ti in the α‐phase.

**Figure 3:**
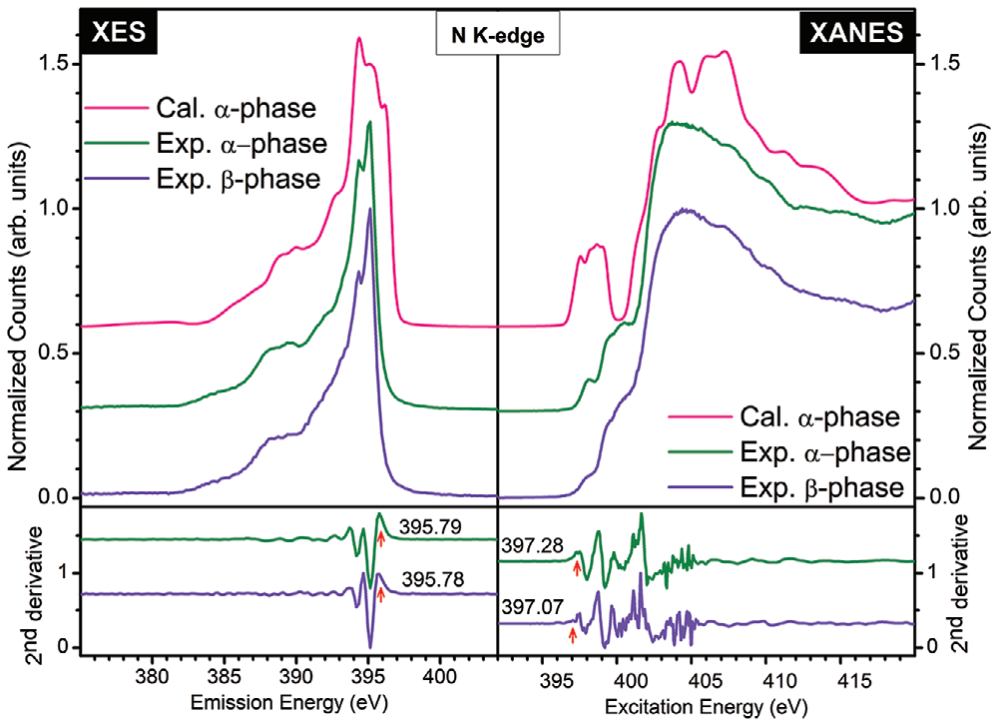
Nitrogen K‐edge emission spectra on the left and absorption on the right panel for α‐ (calculated: magenta and measured: green) and β‐ (measured: viole line) phases of TiP_4_N_8_. The lower panel shows the second derivatives of the measured XAS and XES spectra. The red arrows indicates the peak used to determine the bandgap, with peaks corresponding to valence band edges and conduction band.

### 2.2 N *K* −Edge Analysis: Lone Pairs and Band Gap

**Figure** **3** shows the measured nitrogen K‐edge XAS and XES data for α‐ and β‐phases, illustrating the unoccupied and occupied nitrogen 2*p* projected density of states (DOS) on the same energy scale (shifted by the 1s binding energy). It is important to note that while XAS primarily measures the unoccupied nitrogen 2*p* states, it can also detect titanium 3*d* states due to their hybridization with nitrogen 2*p* states. Therefore, the XAS data reveal the introduction of nitrogen 2*p* states at the bottom of the conduction band near 395 eV for the α‐ and β‐phases. The second derivative of the smoothed XAS and XES spectra is used to estimate the band gap of the β‐phase to be 1.55 ± 0.30 eV, and the α‐phase is 1.77 ± 0.30 eV, respectively with a core hole shifts of 0.1 eV. These values align well with the UV−vis spectroscopy bandgaps reported in previous study.^[^ ^12^ ^]^ In Table S1, we show our calculated bandgap values for mBJ and GGA‐PBE. The 2*p* orbital character in both samples is expected to be similar due to their atomic nature, resulting in comparable charge transfer features (see Figures S4 and S5, Supporting Information). Having similar linear nitrogen bridging interactions in a unit cell of the β‐ and α‐phases, we now focus on the α‐phase.

**Figure 4:**
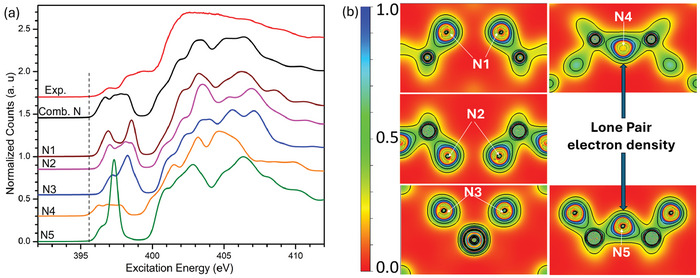
a) Measured and calculated N K‐edge XAS spectra for the α‐phase. The experimental spectra (red) and the combined calculated spectra (black). Colors indicate contributions from different sites N1 through N5. b) The electron density of the α‐phase in the (001) plane for linear (N4, N5) and tri‐planar (N1, N2, N3) nitrogen sites with an isosurface level value of 0.25 eV. This plane shows indirect interaction in the N4 and N5 sites.

**Figure** **4**a shows the measured N K‐edge (promotion of N 1s to the conduction band) and the calculated N spectra across the five nitrogen inequivalent sites of the α‐phase. The calculated spectra are generated by multiplying the pDOS with a dipole transition matrix element and radial transition probability. They are then broadened using Lorentzian and Gaussian line shapes for instrumentation and lifetime effects. Comparing the measured (red) and combined calculated XAS spectrum (black), we can see excellent agreement regarding the energy positions and features. Note that all five inequivalent nitrogen sites contribute to the XAS spectra, with a noticeable lower energy shift observed, especially in the N4 and N5 sites. The onset energies of these sites correspond to the conduction band onset of TiP_4_N_8_. The counts and width of their onset energy indicate that N4 and N5 predominantly contribute to the XAS onset and hence the band gap.

Figure 4b shows a contour map of the electron density for the α‐phase (see Figure S2, Supporting Information, for the β‐phase) in the (001) plane. The outer contour outlines the atom configuration in the selected plane. The electron density at the core of each N1−N3 inequivalent site remains almost spherical. Still, the outer regions of the N4 and N5 sites deviate, extending towards the center down and resulting in an increased electron density (where the lone pair is located). This slight distortion in the nearly spherical density of the N4 and N5 sites is assigned to the indirect interaction of their lone electron pair^[^ ^29^, ^30^ ^]^ with the Ti having a vacant 3*d* orbital. This interaction enhances the mobility of charge carriers such as electrons and holes by improving their delocalization. It reduces recombination rates of the electron‐hole pairs, thus improving the overall efficiency of charge separation and transport within TiP_4_N_8_. As a result, this interaction significantly contributes to the photovoltaic performance by ensuring efficient carrier mobility and reduced losses. It is important to note that the distance of the lone pair to the empty 3*d* orbital (interaction) determines the stability of the β‐ (metastable) and α‐ (stable) of TiP_4_N_8_. In the α‐phase (stable), the lone pairs on N4 and N5 are closer to the titanium atoms, at a distance of ≈2.5 Å. This proximity enhances the interaction strength, stabilizing the structure by improving the coupling between lone pairs and the titanium 3*d* orbitals. The shorter Ti–N bond lengths in the α‐phase (2.0‐2.3 Å) reflect the dominance of Ti^4 +^ ions, which stabilize the lattice due to their smaller ionic radius. In contrast, the β‐phase (metastable) exhibits a weaker interaction as the lone pairs are farther from the titanium atoms ( 3.1 Å). The larger Ti–N bond lengths, combined with the higher presence of Ti^3 +^ ions, lead to reduced lattice stability.

Concerning neutrality, TiP_4_N_8_ remains electrically neutral due to the indirect interaction of linear nitrogen bridging across the larger faces of titanium polyhedral structures. These linear nitrogen bridges, which span across the larger faces of the polyhedra, have lone electron pairs. Thus, the extensive network of linear nitrogen bridges across the larger polyhedral faces ensures that the charge imbalance caused by Ti^+3^ is effectively neutralized, maintaining the overall electrical neutrality of the TiP_4_N_8_.

The Ti^3 +^ ions introduce one additional valence electron compared to Ti^4 +^ ions. This extra electron is primarily localized on the N5 atom, which is within the first coordination sphere ( 2.5 Å from Ti1). This localization strengthens the bonding interaction and stabilizes the electronic structure. The predominance (91.9%) of Ti^4 +^ ions leads to shorter Ti–N bond lengths, which compact the structure and further stabilize the α‐phase. The smaller ionic radius of Ti^4 +^ compared to Ti^3 +^ ions ensures a robust and tightly bound lattice.

## 3 Conclusion

In this study, we explore the electronic structure and photovoltaic properties of novel titanium nitridophosphate (TiP_4_N_8_) using a combination of advanced spectroscopic techniques and theoretical calculations. Our findings highlight the significant role of linear nitrogen bridging and its impact on the band gap and electronic states. Through XAS, XES, and RIXS, we map the unoccupied and occupied electronic states, revealing critical insights into the oxidation states and *dd* transitions. The RIXS measurements demonstrate distinct *dd* transitions and charge transfer excitation, which are further corroborated by LFM calculations, confirming the valence state of Ti ion in the β‐phase to be trivalent and mixed trivalent (9.1%) and tetravalent‐valence (90.9%) states in the α‐phase This finding deviates from the formal oxidation of solely tetravalent Ti ion reported by Eisenburger et al.^[^ ^12^ ^]^ Also, LFM calculations on the α‐TiP_4_N_8_, emphasized the strong role of the Ti^+3^ in reproducing the *dd* excitation low energy loss features. The reduced bandgap measurements underscore TiP_4_N_8_ potential in solar energy applications. Specifically, the measured bandgap of 1.55 ± 0.30 eV and 1.77 ± 0.30 eV for the β‐ and α‐TiP_4_N_8_ phases, respectively, falls within an optimal range for photovoltaic activity. These values suggest that TiP_4_N_8_ has semiconductor properties rather than those of a conventional insulator, indicating its potential efficiency in solar energy conversion. We show that the indirect interaction of nitrogen atoms N4 and N5 is pivotal in the onset of the conduction band determines the bandgap range, also enhancing TiP_4_N_8_ light absorption capabilities and stability. We find that the presence of lone pairs interaction with the titanium 3*d* orbitals offers electronic variability (band gap, charge carrier mobility, and photovoltaic performance) and stability for applications of this material.

## Conflict of Interest

The authors declare no conflict of interest.

## Supporting information

Supporting Information

## Data Availability

The data that support the findings of this study are available in the supplementary material of this article.
