# Supplementary material for: Lone Pair and Unique N‐Bridging of Novel Titanium Nitridophosphate
Source: Adv Sci (Weinh). 2025 Feb 11;12(13):2412830. doi: 10.1002/advs.202412830 (PMC11967769; doi:10.1002/advs.202412830)
Supplement: Supplementary file 1 — Supporting Information [file ADVS-12-2412830-s001.pdf]

ADVANCED SCIENCE
Open Access

Supporting Information

for *Adv. Sci.*, DOI 10.1002/advs.202412830

Lone Pair and Unique N-Bridging of Novel Titanium Nitridophosphate

Peter Ufondu, Sakshi, Teak D. Boyko, Monika M. Pointner, Wolfgang Schnick and Alexander Moewes*

Supplementary information: Lone Pair and Unique N-bridging of Novel Titanium Nitridophosphate

Peter Ufondu,[1,*] Sakshi,[1] Teak D. Boyko,[2] Monika M. Pointner,[3]
Lucien Eisenburger,[3] Wolfgang Schnick,[3] and Alexander Moewes[1]

[1]*Department of Physics and Engineering Physics, University of Saskatchewan, 116 Science Place, Saskatoon S7N 5E2, Saskatchewan, Canada*
[2]*Canadian Light Source, 44 Innovation Blvd, Saskatoon, S7N 2V3, Saskatchewan, Canada*
[3]*Department of Chemistry, University of Munich (LMU), Butenandtstraße 5–13, 81377 Munich, Germany*

I. MEASUREMENTS AND TECHNIQUES

A. Experimental Section

The samples were affixed to the sample holder by pressing them into indium foil (freshly scraped to remove surface oxide). All XAS, XES, and RIXS spectra were collected at the REIXS beamline at the Canadian Light Source (CLS). Our soft X-ray absorption spectroscopy (XAS) techniques adequately capture surface and bulk characteristics without additional techniques, and it probe the occupied and unoccupied partial density of states. With the availability of the XAS technique, we obtain comprehensive information about TiP_4N_8 surface and bulk properties. Specifically, the total electron yield (TEY) provides surface-sensitive information, and the total fluorescence yield (TFY) delivers bulk-sensitive data. An additional third absorption technique, inverse partial fluorescence yield (iPFY), is free of self-absorption effects[1], ensuring results closer to the true absorption. By measuring and comparing all three, we have confirmed that all these methods yield consistent material properties (see Fig. S1), reinforcing our analysis's robustness. The XAS shown here is collected using TEY and is normalized using the drain current from a gold mesh upstream of the sample. The XES and RIXS spectra were collected using a Rowland-type grating spectrometer with an effective resolution of 250 meV of the full width at half maximum (FWHM). The XES and RIXS spectra were calibrated using several elastic scatter peaks on the stainless steel sample holder. All measurements were performed in an ultra−high vacuum (1.0×10^{-9} Torr) with the relative angle between the spectrometer and the incoming beam at 90°. The incoming beam was 62.5° from the sample surface.

B. Theory and Statistical Analysis

The analysis of the titanium $L_{2,3}$-edges and nitrogen K-edge was carried out using the ligand field multiplet (LFM) and DFT calculations. In the LFM, to model Hamiltonian,[2], we use the D_{3h} point group and crystal field distortion strength ($D\mu$ and $D\nu$) to describe the splitting of the five degenerate $3d$ orbitals. The Slater integrals (F_{dd}, F_{pd} and G_{pd}) and spin-orbit coupling (ζ_{2p} and ζ_{3d}) are scaled between 80−100% and 90−102%, respectively, of their atomic Hartree-Fock values. [3–5] The initial energy values for the charge transfer energy (Δ), Mott-Hubbard parameters for the Coulomb repulsion (U_{dd}), and the Coulomb attraction for the $2p$ core hole and the $3d$ orbital (U_{pd}) are from the work of Matsubara et al. [6]
The LFM Hamiltonian is computed using Quanty,[7–10] a software package for simulating $3d$ and $4f$ electron systems.
During the simultaneous XAS and RIXS calculations, a Powell fitting algorithm[11] is used to optimize the fitted parameters, and a Gaussian profile of 0.25 eV (FWHM) is used to broaden the final calculated spectra to account for the experimental resolution. DFT calculations of the N K-edge were performed using the WIEN2K software package, which uses full potential and linearized-augmented plane waves with local orbitals to solve the Kohn-Sham equations[12] self-consistently. For most calculations, the generalized gradient approximation of Perdew, Burke, and Ernzerhof (GGA-PBE) was used for the exchange and correlation energies.[12, 13] The total energies are set to converge with an accuracy of 10^{-6} Ry, with a k-mesh of $7 \times 12 \times 7$ for a cell. The ground state calculations are most appropriate for describing the X-ray emission spectra and the density of states (DOS).

*Electronic address: peter.ufondu@usask.ca

C. Codes and Parameters

Source of the Quanty LFM code git repository [14]. The Hamiltonian for the system is set up in the Lua script for Quanty [7–10], encompassing crystal field, spin-orbit coupling, electron-electron interactions, and hybridization parameters. A Python script conducts parameter fitting, adjusting hybridization parameters to match calculated and measured spectra. This involves running Quanty simulations with initial guesses, comparing simulated and experimental spectra, and using the Powell fitting algorithm for iterative parameter adjustment. The goal is to minimize the difference between calculated is and experimental spectra.

II. DATA TABLES AND FIGURES

TABLE S1: *Calculated bandgap with modified Becke-Johnson (mBJ) and generalized gradient approximation Perdew-Burke-Ernzerhof (GGA-PBE). UV-Vis is the measured bandgap in reference[15], and XES−XAS the measurement within this article.*

Sample	Experiment		Calculation	
	UV-Vis (eV)	XES − XAS (eV)	mBJ (eV)	GGA-PBE (eV)
β-phase	1.60	1.55±0.3	–	0.71
α-phase	1.80	1.77±0.3	2.11	1.44

We also compute the bandgap of 1.435 eV for the α-phase using DFT+U, which is the same as that of the GGA potential in Table S1.

TABLE S2: *Broadening factor with Gaussian 0.25 eV.*

Ion		XAS					RIXS	
Ti^{+3}	Position (eV)	457.2	458.5	–	463.4	464.5	0.0	−6.0
	Lorentzian (eV)	0.7	0.3		0.8	0.4	0.2	0.6
Ti^{+4}	Position (eV)	458.0	458.4	460.2	463.5	465.4	0.0	−6.0
	Lorentzian (eV)	0.1	1.0	0.03	1.0	0.9	0.2	0.6

TABLE S3: *The optimized parameters obtained from LFM β- and α-TiP_4N_8 calculations.*

Parameter	Ti^{3+}		Ti^{4+}	
	Scale (%)	β (eV)	Scale (%)	α (eV)
$D\mu$	83	0.300/0.500	99	−0.368
$D\nu$	17	−0.0709	99	−0.247
ζ_{2p}	100	3.710	90	3.776
ζ_{3d}	90	0.019	100	0.019
$X\zeta_{3d}$	90	0.027	91	0.032
$F2_{dd}$	99	5.907	92	0.000
$F4_{dd}$	99	3.711	92	0.000
$F2_{pd}$	80	3.187	80	6.301
$XF2_{dd}$	95	10.342	80	0.000
$XF4_{dd}$	95	6.499	80	0.000
$G1_{pd}$	80	3.393	80	4.626
$G3_{pd}$	80	1.258	80	2.632
U_{dd}	–	2.000	–	2.425
U_{pd-dd}	–	0.112	–	2.000
10_{DqL}	–	0.010	–	0.987
Δ	–	3.900	–	5.567
V_{a1}	100	1.425/1.026	100	0.750
V_{e1}	100	−0.929/−0.529	100	0.985
V_{e2}	100	−0.783/−0.583	100	−0.623

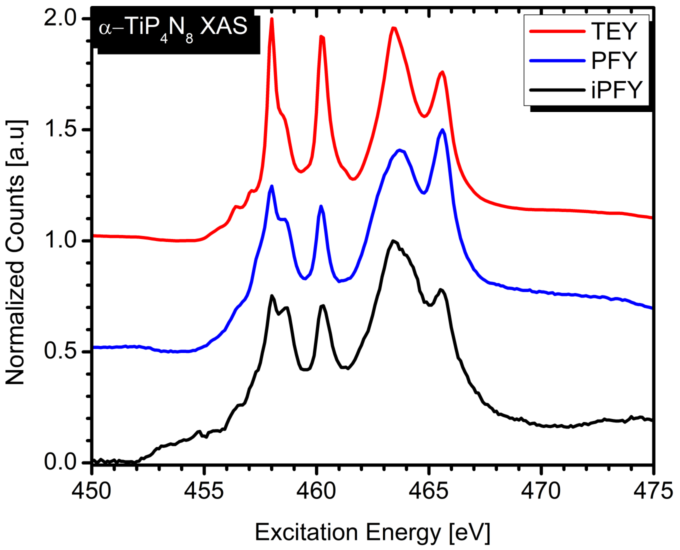

FIG. S1: *X-ray absorption spectra (XAS) at the Ti $L_{2,3}$-edges of α-TiP_4N_8 obtained using total electron yield (TEY), partial fluorescence yield (PFY), and inverse partial fluorescence yield (iPFY). The spectra, plotted as normalized counts versus excitation energy (eV), show strong similarity across all methods, indicating consistent results regardless of the detection approach. TEY primarily probes the surface of the sample, while PFY and iPFY provide bulk-sensitive measurements. iPFY, derived from the inverse of the fluorescence signal, enhances sensitivity to the bulk by reducing self-absorption effects[1].*

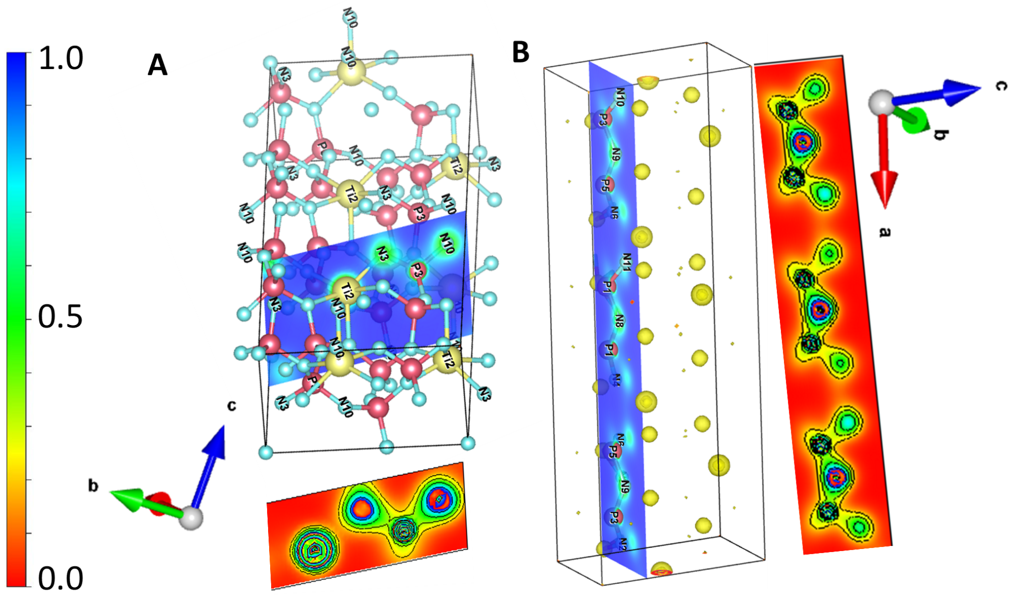

FIG. S2: *Visualization of the electron density distribution in the β-phase, presented as a 3D map (unit cell and slice) and a 2D contour map for the tri-planar (A) and linear (B) configurations. In panel A, the slice is taken along the (-12 1 2) plane, highlighting the electron density maps corresponding to atoms N10 and N3. In panel B, the slice is extracted along the (0 0 1) plane, showcasing the electron density around atoms N9 and N8. The color scale on the left represents the normalized electron density values for the 2D map, with higher densities indicated by the blue regions. The 2D contour maps on the orange planes illustrate the spatial variations in electron density, further emphasizing the differences between the tri-planar and linear bonding and interaction configurations. The axes in each panel define the crystallographic directions, aiding the spatial interpretation of the structures.*

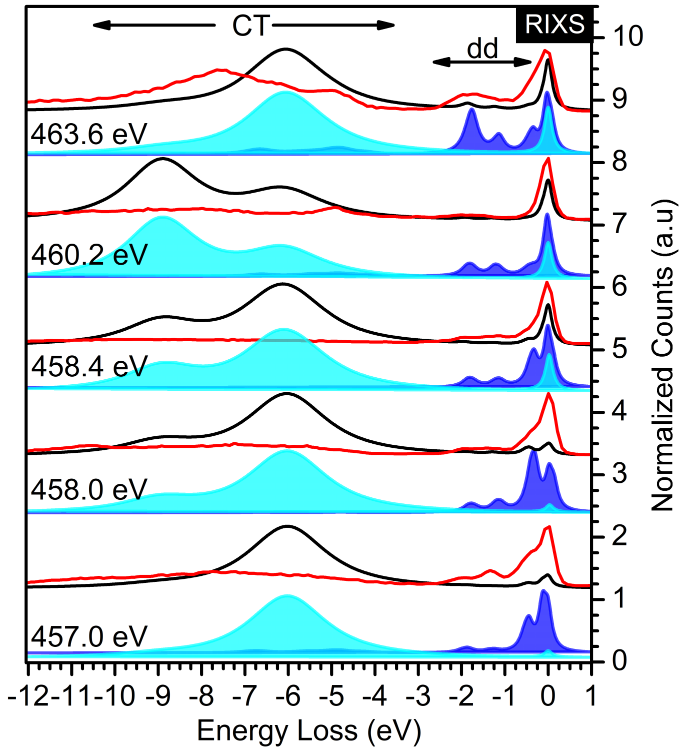

FIG. S3: *The RIXS spectra, both experimental and calculated, exhibit dd excitation and charge transfer excitation in the α-phase. The measured spectra are represented in red, while the combined titanium (Ti^{+3} and Ti^{+4}), Ti^{+3}, and Ti^{+4} are depicted in black, blue, and cyan, respectively.*

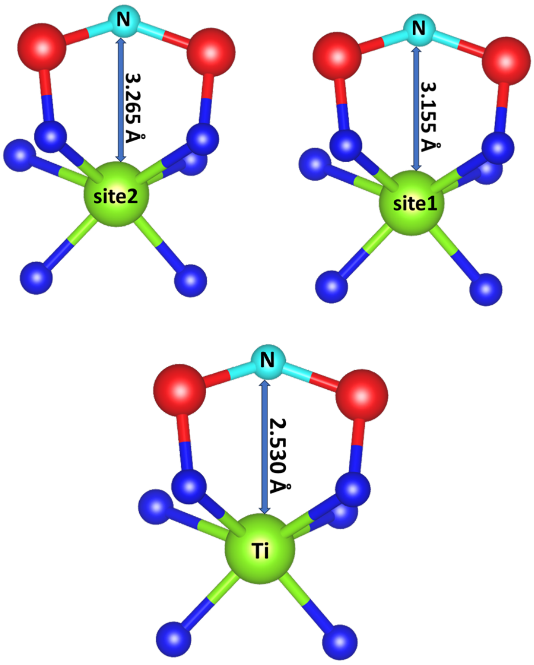

FIG. S4: *Shows the distance of the linear nitrogen bridge on the larger octahedral surface from the titanium center. Top: The two titanium sites in the β−phase (Site 1 and 2). Bottom: The α−phase. The blue and cyan spheres are tri-planar and linear nitrogen atoms, while the red and green spheres are the phosphorous and titanium atoms, respectively.*

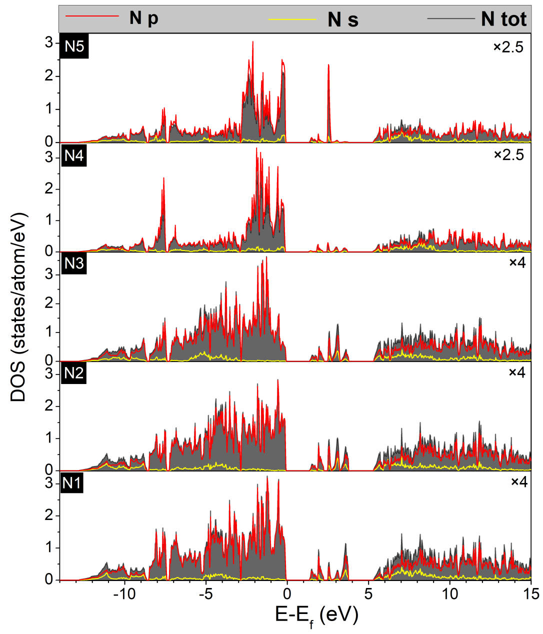

FIG. S5: *The pDOS of the α-phase for all five nitrogen inequivalent sites. The total DOS is represented by the shaded grey region. The p-states are shown in red, and the s-states are in yellow, with each scaled for comparison.*

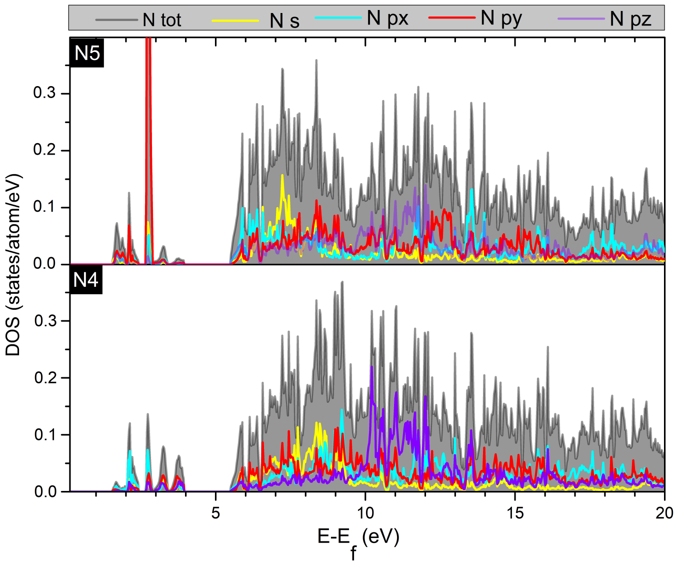

FIG. S6: *The pDOS for N4 and N5 sites for the α-phase. The total DOS for nitrogen on these sites is represented in the shaded grey. The s- and p- states are shown in different colors to illustrate their contribution to the bandgap for N4 and N5 of the α-phase.*

[1] P. M. Braun, R. J. Green, L. Dubrovinsky, S. V. Ovsyannikov, and A. Moewes, Journal of Materials Chemistry C **11**, 10864 (2023).

[2] F. M. F. de Groot and A. Kotani, *Core level spectroscopy of solids* (CRC Press, 2008), ISBN 9781420008425, URL `https://books.google.ca/books?id=HGHzu66i1yoC`.

[3] J. Slater and J. Slater, *Quantum Theory Of Atomic Structure Vol 1-2.*, no. V. 11 in International series in pure and applied physics (McGraw-Hill, 1960), URL `https://books.google.ca/books?id=iSzrxAEACAAJ`.

[4] F. M. F. de Groot, J. C. Fuggle, B. T. Thole, and G. A. Sawatzky, Physical Review B **42**, 5459 (1990), URL `https://link.aps.org/doi/10.1103/PhysRevB.42.5459`.

[5] R. J. Green, V. Zabolotnyy, M. Zwiebler, Z. Liao, S. Macke, R. Sutarto, F. He, M. Huijben, G. Rijnders, G. Koster, et al., Physical Review Materials **5**, 065004 (2021), URL `https://link.aps.org/doi/10.1103/PhysRevMaterials.5.065004`.

[6] M. Matsubara, T. Uozumi, and A. Kotani, Journal of Synchrotron Radiation **8**, 393 (2001), URL `https://onlinelibrary.wiley.com/doi/abs/10.1107/S0909049500016538`.

[7] M. W. Haverkort, M. Zwierzycki, and O. K. Andersen, Physical Review B **85**, 165113 (2012), URL `https://link.aps.org/doi/10.1103/PhysRevB.85.165113`.

[8] Y. Lu, M. Höppner, O. Gunnarsson, and M. W. Haverkort, Physical Review B **90**, 085102 (2014), URL `https://link.aps.org/doi/10.1103/PhysRevB.90.085102`.

[9] M. W. Haverkort, G. Sangiovanni, P. Hansmann, A. Toschi, Y. Lu, and S. Macke, Europhysics Letters **108**, 57004 (2014), URL `https://dx.doi.org/10.1209/0295-5075/108/57004`.

[10] M. W. Haverkort, Journal of Physics: Conference Series **712**, 012001 (2016), URL `https://dx.doi.org/10.1088/1742-6596/712/1/012001`.

[11] M. J. D. Powell, The Computer Journal **7**, 155 (1964), ISSN 0010-4620, URL `https://doi.org/10.1093/comjnl/7.2.155`.

[12] K. Schwarz, P. Blaha, and G. Madsen, Computer Physics Communications **147**, 71 (2002), ISSN 0010-4655, proceedings of the Europhysics Conference on Computational Physics Computational Modeling and Simulation of Complex Systems, URL `https://www.sciencedirect.com/science/article/pii/S0010465502002060`.

[13] J. P. Perdew and K. Schmidt, AIP Conference Proceedings **577**, 1 (2001), ISSN 0094-243X, https://pubs.aip.org/aip/acp/article-pdf/577/1/1/12108089/1_1_online.pdf, URL `https://doi.org/10.1063/1.1390175`.

[14] C. M. Meier, P. M. Braun, and P. Ufondu, *QuantyLF* (2024), gitHub repository, URL `https://github.com/CMM02/QuantyLF`.

[15] L. Eisenburger, V. Weippert, C. Paulmann, D. Johrendt, O. Oeckler, and W. Schnick, Angewandte Chemie International Edition **61**, e202202014 (2022), https://onlinelibrary.wiley.com/doi/pdf/10.1002/anie.202202014, URL `https://onlinelibrary.wiley.com/doi/abs/10.1002/anie.202202014`.
